# Electronic Brief Intervention and Text Messaging for Marijuana Use During Pregnancy: Initial Acceptability of Patients and Providers

**DOI:** 10.2196/mhealth.7927

**Published:** 2017-11-08

**Authors:** Justin Gray, Jessica R Beatty, Dace S Svikis, Karoline S Puder, Ken Resnicow, Janine Konkel, Shetoya Rice, Lucy McGoron, Steven J Ondersma

**Affiliations:** ^1^ Merrill Palmer Skillman Institute Department of Psychiatry and Behavioral Neurosciences Wayne State University Detroit, MI United States; ^2^ Department of Psychology Virginia Commonwealth University Richmond, VA United States; ^3^ Department of Psychiatry Virginia Commonwealth University Richmond, VA United States; ^4^ Department of Obstetrics and Gynecology Virginia Commonwealth University Richmond, VA United States; ^5^ School of Medicine Department of Obstetrics and Gynecology Wayne State Unviersity Detroit, MI United States; ^6^ School of Public Health University of Michigan Ann Arbor, MI United States

**Keywords:** pregnancy, marijuana, intervention study, text messaging

## Abstract

**Background:**

Marijuana is the most widely used illicit substance during pregnancy. Technology-delivered brief interventions and text messaging have shown promise in general and pregnant samples but have not yet been applied to marijuana use in pregnancy.

**Objective:**

The objective of the study was to evaluate, among pregnant women and prenatal care providers, the acceptability of an electronic brief intervention and text messaging plan for marijuana use in pregnancy.

**Methods:**

Participants included patients (n=10) and medical staff (n=12) from an urban prenatal clinic. Patient-participants were recruited directly during a prenatal care visit. Those who were eligible reviewed the interventions individually and provided quantitative and qualitative feedback regarding software acceptability and helpfulness during a one-on-one interview with research staff. Provider-participants took part in focus groups in which the intervention materials were reviewed and discussed. Qualitative and focus group feedback was transcribed, coded manually, and classified by category and theme.

**Results:**

Patient-participants provided high ratings for satisfaction, with mean ratings for respectfulness, interest, ease of use, and helpfulness ranging between 4.4 and 4.7 on a 5-point Likert scale. Of the 10 participants, 5 reported that they preferred working with the program versus their doctor, and 9 of 10 said the intervention made them more likely to reduce their marijuana use. Provider-participants received the program favorably, stating the information presented was both relevant and important for their patient population.

**Conclusions:**

The findings support the acceptability of electronic brief intervention and text messaging for marijuana use during pregnancy. This, combined with their ease of use and low barrier to initiation, suggests that further evaluation in a randomized trial is appropriate.

## Introduction

A growing literature suggests that marijuana use during pregnancy is associated with low birth weight, stillbirth, and neurologic or developmental impairment [1-4]. Subtle sequelae are most apparent later in development, with impaired cognition, decreased academic achievement, and increased risk of abusing drugs later in life being the most prevalent [5]. Despite these possible adverse outcomes, marijuana use during pregnancy often goes unaddressed. Physicians and other providers must act with caution when considering drug use, especially marijuana, in a pregnant population. This has become increasingly important because past month marijuana use in the general population (22.2 million Americans, aged 12 or older) has increased from 6.2% in 2002 to 8.3% in 2015, with 19.8% (6.8 million) of those aged between 18 and 25 reporting past month use [6]. Among pregnant women, 6.4% of those in the age group of 18 to 25 years report past month use, and 1.3% of those aged between 26 and 44 years report past month use [7]. An increasing number of patients are turning to this substance for its antiemetic properties; nausea is an approved indication in all states where medical use of this drug has been legalized [8]. This puts an increasing number of pregnancies at risk.

Women with substance use disorders are less likely to enter treatment than men [9]. Furthermore, most persons in need of treatment neither receive it nor feel that they need it [10], suggesting a need for approaches that are brief enough to be acceptable even to those who are unwilling to engage in formal treatment. As a consequence, screening, brief intervention, and referral for treatment (SBIRT) has become a recommended core element of prenatal care [11].

However, integration of SBIRT into the clinical setting carries with it several practical challenges. Evidence from attempts to implement SBIRT for alcohol and tobacco have demonstrated large variations in efficacy, significant SBIRT-related training costs, and a notable increase in overall visit length [12,13]. Furthermore, additional physician and staff training in SBIRT focusing on smoking cessation has been shown to have modest or transient effects on trainee behavior, and few prenatal care providers fully implement recommended brief intervention strategies [14-17]. In one national survey, only 27.49% of women (1323 out of 4812) reported even being asked about alcohol use by their primary care provider [18]. These challenges—time, burden, and training-related—are only magnified when seeking to add additional screening and brief intervention foci such as marijuana on top of existing calls to address alcohol and tobacco. Furthermore, disclosure of substance abuse can be both socially stigmatizing and subject to potential legal consequences (at least in most states; it remains to be seen whether use in pregnancy will be viewed differently in states where it is legalized, as women are most often pursued via child welfare statutes rather than on drug-related charges). Regardless, this stigmatization and social risk suppresses disclosure, which in turn further limits the proportion of at-risk women who receive even a brief intervention.

Electronic administration of SBIRT (eSBIRT) may help to address these challenges. Computer and electronic approaches could potentially reduce financial barriers, increase reproducibility, and facilitate consistent delivery across patient populations [19]. These approaches also offer the ability to tailor parts of the program based on patient responses, conferring a feeling of personal relevance to participants that enhances message impact [20]. In addition, computer-delivered screening is associated with greater disclosure of drug use, as is provision of anonymity [21], which is possible via technology-delivered brief interventions.

Delivering SBIRT via technology also allows incorporation of text messaging, which can readily use data collected during screening and brief intervention to send subsequent tailored communications. Text messages have a number of advantages, including the near ubiquity of mobile phone ownership: 100% of adults in the age group of 18 to 29 years own a cell phone, and, as of 2016, 100% of people aged between 18 and 29 years who own a cell phone use it for texting at least occasionally [22]. Text messaging interventions carry relatively low operational costs that do not increase as the reach of the program increases [23]. Furthermore, a recent systematic review found that text messages were particularly effective as a supplement to Internet-based interventions [24]. This result may reflect some of the relative advantages of text messaging such as (1) the ability of text-based messaging to maintain multiple communications with a participant without reliance on a subsequent meeting; (2) the ability of text messages to reach the participants in their natural environment; and (3) the fact that text messages—unlike other forms of communication, such as mailings—are nearly always opened (99% are opened, 90% within the first 3 min of receipt [25]). For example, with regard to smoking in pregnancy, Naughton et al (2011) found that participants receiving tailored SMS (short message service) text messages were more likely to set a quit date and reported higher levels of self-efficacy, harm beliefs, and determination to quit than their control counterparts [26]. Additionally, Quit4baby (Voxiva Inc), a text message program designed to help women reduce tobacco use during pregnancy, was rated as helpful in getting them to make a quit attempt and providing ideas on ways to quit [27].

Text messaging and eSBIRT may therefore have utility in addressing marijuana use in pregnancy. However, neither of these approaches has previously been tested in this context. This study is a preliminary, qualitative, and quantitative examination of the acceptability of eSBIRT and a tailored texting protocol among 2 key samples: (1) pregnant women reporting regular marijuana use during the month before becoming pregnant and (2) prenatal clinic staff. Qualitative and quantitative feedback described in this study informed changes before the start of a larger clinical trial.

## Methods

### Participants

#### Patient-Participants

Patient-participants were 10 women recruited from a prenatal clinic in Detroit, Michigan. Inclusion criteria were self-report of marijuana use at least twice weekly in the month before pregnancy, aged between 18 and 40 years, less than 20 weeks pregnant, and owning a cellphone (As participants would be responsible for any charges resulting from receiving text messages on their personal phone, all participants were specifically asked their willingness to receive text messages during a feedback interview.). Exclusion criteria included inability to understand English, inability to provide consent, consideration of an elective abortion or adoption for the current pregnancy, or past participation in any other study by the authors.

#### Provider-Participants

Of the medical staff from the same prenatal clinic, 12 members volunteered to participate in focus groups regarding the intervention materials. We offered participation to all physicians in the department; 5 physicians were available to attend one focus group, and 7 medical staff (all of the nurses, medical assistants, and reception staff from the clinic where recruitment took place) participated in the second focus group.

### Procedure

Recruitment for pregnant participants began on March 10, 2015, and ended on July 10, 2015. In total, 61 participants were screened over 41 days of recruitment to obtain the 13 (21%) eligible participants. Of the 13 eligible patients, 10 (77%) participated in the feedback session. Participants were recruited until saturation was reached (high quantitative ratings of the intervention’s ease of use, usefulness, and attitude toward using the intervention, and no new information obtained from interviews) [28]. Focus group data were collected during March 2015.

Patient-participants were given a flyer by clinical staff describing the study. Medical staff explained that it was a voluntary research project involving 5 to 10 min to determine eligibility for a larger study and that they would receive a small gift for their baby if they choose to participate. Those showing interest were introduced to the research assistant, who prescreened for age, gestation, ability to understand spoken English, and ability to receive text messages. Women passing this prescreen process reviewed an informed consent information sheet on a study provided on a tablet personal computer (PC) with headphones (with disposable sanitary covers). Those who consented completed eligibility screening.

Interested and eligible patient-participants reviewed a second research information sheet approved by the institutional review board (IRB) describing the anonymous, single-session study. Only patient-participants who were eligible were told that the study was focused on reducing marijuana use during pregnancy. Those who provided consent to participate spent approximately 1 hour reviewing the intervention materials on the tablet PC and completing a semistructured feedback interview (approximately 30 min). All patient-participant activities were conducted via individual sessions with research staff in a private office within the clinic.

All patient-participants responded to qualitative acceptability items using the same tablet PC used for consent and intervention delivery. The research assistant conducted the semistructured interview after patient-participants completed the intervention and acceptability items. The brief interview was designed to elicit the participant’s overall impression of the software, evaluations of its helpfulness, likes and dislikes, and suggested changes. Unclear or short responses were probed to elicit more information. Once questions about the intervention and videos (included as part of the intervention) were answered, participants were provided with a list of text messages to review. It was explained that these messages would be sent on a schedule selected by the patient (ie, once, twice, or 3 times a week) over the course of their pregnancy. Patient-participants were told that those participating in the later clinical trial would receive text messages that were tailored based on the responses provided during the initial screening and software intervention. For example, when asked whether they had ever been prescribed medication for depression, anxiety, or any other emotional difficulties, patient-participants would receive one of two messages. If they answered in the affirmative, the following message would be sent:

Smoking may seem to help when you are feeling down, but after, it may make you feel worse. Pay attention to your body and emotions. Talk to your doc for help facing your struggles.

If patients denied ever seeking outside services, they would receive the following message:

Every day you don’t smoke, be extra good to yourself. You deserve it. Tell yourself, “What I am doing is amazing.”

The research assistant pointed out this single example of how text messages could be tailored before allowing the participant to review the list. After the participants reviewed the text message list, they completed the software acceptability items and were subsequently interviewed.

The research assistant received training in conducting semistructured interviews from one of the coauthors (JRB) and was supervised for several practice interviews to determine competency. All responses were transcribed verbatim during the interview for later analysis. A total of 39 questions were asked of all participants. After completion of the interview, all participants were provided with a referral guide, encompassing multiple areas of risk (eg, substance abuse, emotional health, and education and job training), for relevant services in the area. Procedures were in place for active referral to clinic staff and local treatment centers, but none of the participants expressed interest in this level of service at the time of the interview. Participants received a US $30 Target gift card for participation in the study.

The provider-participant focus groups were conducted on two different dates: one with physicians and one with other medical staff. Procedures were the same for both focus groups. An informed consent information sheet was reviewed before each focus group began. Medical staff were then shown the intervention on a large projector screen. Initial reactions were obtained halfway through the intervention and again after the end of the intervention. After all interview questions were answered, staff were handed a copy of the potential text messages and asked for reactions related to that information. Before concluding the focus group, all staff were asked about the best ways to implement the study without disrupting the flow of ongoing care. Each focus group was audio-recorded and then transcribed. All procedures were reviewed and approved by the university IRB before any recruitment.

### Measures

#### Software Acceptability (Patient-Participants)

Acceptability of the computer-delivered intervention was assessed using participant responses to 12 self-report questions (6 additional questions focused on the videos shown as part of the brief intervention and 10 on text messages). These 28 items, relating to ease of use, respectfulness, helpfulness, and likability, were rated using a 5-point Likert scale (with 1=not at all and 5=very much) after completion of the intervention. The question, “How much did some parts of the computer bother you?,” was reverse-coded, with 1=very much and 5=not at all. These acceptability questions were similar to those developed and used successfully in previous usability testing [29] and are based on the Technology Acceptance Model [30]. Additionally, 1 yes-no question asked participants about change likelihood as a result of the intervention:

Are you more likely to be successful with this goal [of quitting marijuana] because of your participation here?

#### Feedback Interview (Patient-Participants)

The 39-item open-ended interview used in this study was based on expert opinion (the majority of the coauthors were included in this group) and consensus on what information would be helpful in making modifications to the intervention. See Textbox 1.

#### Focus Group Interview (Provider-Participants)

Focus group questions focused on how the providers felt their patients would react to the intervention materials, how helpful they felt the intervention materials were, and how the study procedures could be best integrated into the ongoing clinic procedures. The questions were similar in language and content to those asked of the patient-participants.

### Interventions

The brief intervention developed for this study uses patient responses to provide an individualized, interactive experience. A three-dimensional animated narrator guides the participant through the intervention. The narrator is able to speak, move, provide empathic reflections, and display appropriate emotional responses. The program includes aural as well as visual presentation of all content, and all answers are recorded by simply tapping responses from a list or by touching a visual analogue scale. The narrator reads aloud any written material on the screen, including response options (participants just have to click on the word or phrase to hear it read aloud).

**Figure 1:**
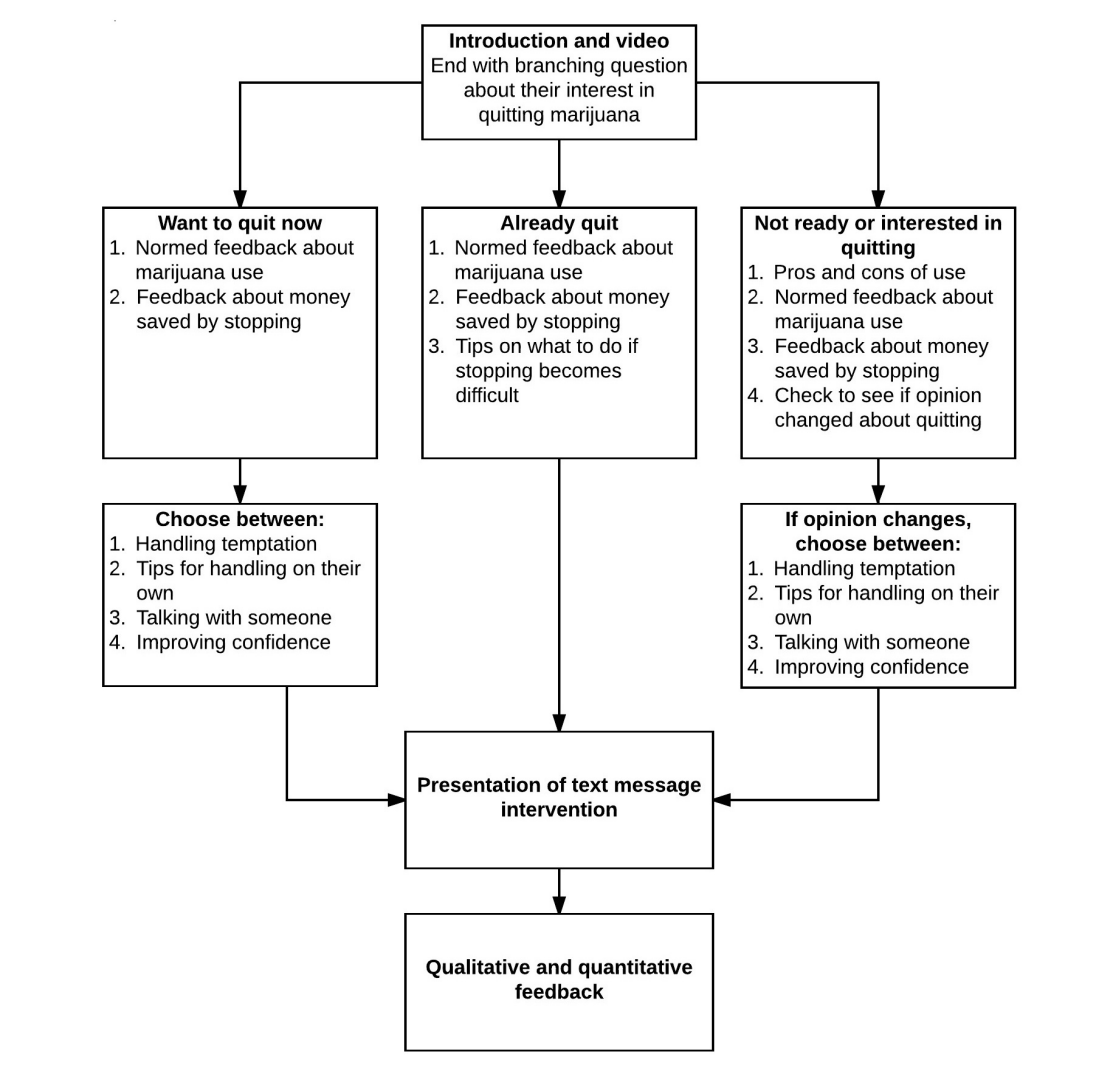
Intervention flow.

The intervention content was adapted from brief intervention [31-33] and motivational interviewing techniques [34]. It was adapted and modified to specifically address marijuana use from a previous brief intervention designed to reduce alcohol use during pregnancy [35]. The intervention begins with a brief introduction, followed by an embedded video of a physician discussing potential benefits of reducing or quitting marijuana use during pregnancy and of a mother describing her own decision to quit using marijuana while pregnant (all were actors). Next, participants are asked to report how they feel about their use of marijuana while pregnant. Those who reported already stopping all marijuana use receive normed feedback designed to reinforce the decision to stop using, were asked to provide the reasons and advantages for their decision to quit, and were helped to develop a personalized plan for preventing relapse. This branch of the intervention was designed to be potentially efficacious with both women who have quit and women who have not quit but choose to indicate abstinence to avoid negative reactions. Participants who endorsed active use received content that was consistent with traditional brief intervention approaches and that included normed feedback (normed for age, pregnancy status, and gender), decisional balance exercises, and an optional change plan with a menu of change options. See Figure 1 for a visual diagram of the intervention flow.

The video embedded in the intervention featured a physician providing gain-framed information about the benefits of reducing marijuana use during pregnancy and a mother providing a testimonial regarding her decision to avoid marijuana while pregnant. Multiple versions of each video were available and were tailored to participants based on self-reported self-efficacy, race, and motivation.

The text messages were designed to be tailored on a number of factors, including self-efficacy, gestational stage, social support, and processes of change based on the theory of planned behavior [36] and self-determination theory [37]. Approximately two-thirds of messages are related to the participant’s specific goal regarding marijuana: the remaining one-third of the content was designed to provide either community resources relevant to the pregnancy or inspirational quotes. All intervention materials were developed with ongoing expert feedback and final review before being presented to participants.

**Interview questions.**

Intervention

1. What did you think about the program?
2. What parts did you like the most?
3. What parts did you like the least?
4. What, if anything, do you think should be changed?
5. What do you think about the introductions?
6. How was [the narrator’s] voice?
7. Did any of the questions of parts of the program bother you?
8. What was useful to you in the program?
9. How has using it changed your thoughts on your marijuana use, if at all?
10. Did you make a personal plan for how to change your marijuana use?

Video

1. What did you think about the videos?
2. What bothered you about the videos?
3. What did you like about the videos?
4. What could we do to improve the videos?
5. Did the videos give you the impression that we didn’t understand marijuana or that we were acting like it’s more dangerous than it really is?
6. Did the video feel preachy or judgmental?
7. What did you learn from the videos?
8. Was the woman someone you could relate to? Why or why not?
9. Did the videos change your opinion in any way?

Short message service

1. What did you think about the text messages?
2. Did any of the content on the messages bother you?
3. What words/language made you uncomfortable?
4. What was useful about the messages?
5. How much did it feel like the messages were intended for you? What could have made them feel more personalized?
6. How did you feel about the advice you were given?
7. How did you feel about the amount of information? Too much or too little?
8. What would you have liked to see added to text messages? Was there anything that didn’t need to be there?
9. How have your feelings about your marijuana changed since you read the messages? What did it make you think about or want to do differently, if anything?
10. Would you be comfortable receiving these messages on your phone?
11. If you got these messages and someone saw them on your phone, how would you feel about that?
12. Do you have a phone with texting abilities? If yes, how often do you use texts?
13. If you were to receive text messages such as these, how often would you like to receive them?

### Data Analysis

Means, standard deviations (SDs), and counts for demographic data and software acceptability ratings were analyzed via SPSS version 22 (IBM Corp). Qualitative data were subjected to manual, thematic coding of participants’ oral responses of transcribed interviews (patient-participants) and focus groups (provider-participants). Responses were grouped by similar patterns or themes that occurred throughout the interviews [38]. Next, coders decided what construct label best described each of the groupings. The coding and classification of the responses were performed and validated independently by 2 members of the investigative team (JG and JRB). Both coders met to compare the independently generated groupings and themes. All disagreements were discussed until a consensus was reached for a total of 10 themes.

## Results

### Quantitative Feedback

Using a 5-point rating scale, average ratings for the intervention were all at or above 4.4, with the highest rating being for excitement about making a change in marijuana use (mean 4.9, SD 0.38; see Table 1). All 10 patient-participants reported feeling more likely to change their marijuana use because of their interaction with the computerized intervention. All ratings for the video were at or above 4.0 except for the item “The mom in the video looks like me” (mean 3.7, SD 1.49). Average ratings of the following text message items were below 4.0: likability (mean 3.7, SD 1.0), how interesting (mean 3.7, SD 1.23), helpfulness (mean 3.6, SD 1.06), got you thinking about your use (mean 3.3, SD 1.41), and interest in receiving text messages (mean 3.2, SD 1.09).

**Table 1:** Quantitative ratings of software, video, and short message service (SMS) acceptability (scored on a 5-point Likert scale, with 1=not at all and 5=very much; positive rating=score of 4 or 5; n=10 for intervention and video ratings, n=9 for text message ratings).

Question	Giving a positive rating, n (%)		
	Intervention	Video	Text messages
Likability	7 (70)	5 (50)	5 (56)
Ease of use	10 (100)	-	8 (89)
How interesting	8 (80)	-	5 (56)
Respectful	9 (90)	-	9 (100)
Bothered by parts of program^a^	8 (80)	-	7 (78)
Helpfulness	9 (90)	-	4 (44)
Feel better about yourself	8 (80)	-	7 (78)
Got you thinking about your use	9 (90)	-	5 (56)
Program seemed to understand you	9 (90)	-	-
Interest in working with program again/receiving messages	7 (70)	-	3 (33)
Excited about changing marijuana use during pregnancy	10 (100)	-	-
Think other moms would be helped	9 (90)	-	7 (78)
Learned new information	-	7 (70)	-
Learned useful information	-	7 (70)	-
More motivated to avoid marijuana	-	9 (90)	-
Already knew information presented	-	6 (60)	-
Mom in video was like me	-	5 (50)	-

^a^This item was reverse-scored.

### Qualitative Feedback

Analysis of open-ended responses yielded 7 major themes among the 3 sections of the software arising from the patient-participant interviews, as well as 3 major themes arising from the medical staff focus feedback group; see Textbox 2.

### Intervention

#### Liked Working With the Program

Of the patient-participants, 8 reported feeling some personal relevance of the information whereas the remaining 2 were ambiguous. When asked whether they would rather talk to the program or a person about their marijuana use, 5 patient-participants indicated that they preferred interacting with the program, whereas 4 said they would have preferred talking to their doctor; one participant reported that either option was fine. For example one patient-participant said:

It’s hard to be truthful with people you don’t know. It wouldn’t feel so awkward as talking with real people you don’t know, it’s hard. It’s easier to tell the whole truth with the computer.

#### Supported or Changed Thoughts About Marijuana

After completing the computerized intervention portion of the study, 6 patient-participants reported making a plan to quit; 3 reported already ceasing use, and one felt she could stop on her own; 7 patient-participants reported being more likely to stop marijuana use after interacting with the program. When asked about the most useful part of the program, 4 women highlighted the tips on how to cut down or quit and 2 mentioned the information about how much money they could save by quitting. All but 2 patient-participants indicated that interacting with the program changed something about how they saw their use of marijuana during pregnancy. For example, another patient-participant said:

It was great because [the narrator] gave true facts about marijuana use. [The narrator] gave better information. It’s better than listening to people on the street.

**Themes and examples.**

Intervention

- Liked working with the program
  - Real great, very educational too.
  - It was well balanced, covered both the good things and bad things and helped me not feel bad about my use.
  - [The program] makes it less awkward. I am okay talking with people I know. [The program] would be more helpful, because the whole truth will come out.

- Supported or changed thoughts about marijuana
  - It boosted my confidence up, like you can do it girl.
  - Standing firm against temptation: I like when [the narrator] said take it out of the house and stay away from people that do it.
  - I actually learned that marijuana may not affect your pregnancy at the moment with an infant, like it could be later on in life.

- Women in the videos were helpful
  - Relatability of actors/actresses used was important to most, if not all, of the participants.
  - The mother’s testimony, I love that part. She told how she got through it by thinking about the baby.
  - More testimonials may hit people in a different way, it can relate to more people.

- More information about harmful effects
  - Dr said marijuana use can cause learning disability, I didn’t know that.
  - The MD’s video made me want to be as healthy as possible to have a healthy baby.
  - More in depth for ladies on the second or third pregnancies. Even though I’m on my fifth pregnancy, I’m pretty sure there is something I need to learn still.

Short message service feedback

- Text messages were helpful.
  - Really good topics, would pass it on to friends.
  - Great idea, a lot of people use cell phones and text.
  - I like them because it’s a new idea and a way to motivate because they can be experiencing a moment of temptation.
  - Helpful...to deal with bad moods and things to do so you won’t relapse.

- Learned about community resources
  - I like the dial 2-1-1 for help.
  - Text messages can help people quit and provide resources that we didn’t know about.

- Information presented was overwhelming
  - Excessive, a lot of information.
  - Too much—make them briefer.
  - A little too much, people will get bored reading all that.

Medical staff focus group

- Concern about patient takeaway
  - Dose does matter. If someone does cut back, that’s a victory too. I think this is good message if low motivation. A stronger message would be needed for high motivation women.
  - Sounds kind of soft in terms of message about marijuana—hedges too much.
  - Last line comes across as if you use less, than that’s okay too. I think I would like an “abstinence” message, but not sure. We’ll take what we can get, but thinks it leave the door open where it’s okay to use a little.

- Integration into patient visit
  - The time after they are “roomed” is wasted. It would give them the opportunity to do something.
  - All the time moms sit [at the office] for glucose testing—would be a good opportunity to catch them or give a second dose of the intervention.

- Quality of the presentation
  - I liked it. It would connect well. A lot of my patients are surrounded by marijuana. I like how the woman [in the video] talks about how she made up her own mind—drug use is kind of rebellious and this allows her to keep this.
  - [Specific text message] should be at the beginning. That’s when everything is nasty. They feel they need weed to eat. A lot of patients feel like that. They have to be told to try other foods.
  - A couple of the [screens] are too busy. Follow the rule of 6—no more than 6 words per line and 6 lines of text.
  - [The doctor in the video] really looks like an OB/GYN instead of an actress; authentic.

### Video

#### Women in the Videos Were Helpful

Relatability of the testimonials was brought up by several patient-participants. Although many were critical of one of the actresses (saying that she wasn’t believable and “seemed directed as if reading off a script”), most reported liking another actress who was African American and who spoke in a casual, unscripted manner. Overall, patient-participants found the testimonials to be helpful, and some found it to be the most helpful element. For example, one patient-participant said:

The mother’s testimony, I love that part. She told how she got through it by thinking about the baby. I tell my friends to think about the baby too. The video boosted my confidence.

#### More Information About Harmful Effects

In addition to asking for more testimonials, patient-participants also asked to see more information about the effects of marijuana on the mother and fetus. Several women stated they would like to hear more of this information from the physician in the video in addition to the patient testimonials. For example, one patient-participant said:

The doctor’s video made me want to be as healthy as possible for my baby.

Information about the real effects of marijuana and about the controversy in this area was also well received. When asked what she liked about the videos, a different patient-participant said:

The picture of the pregnant woman smoking and people on either side saying good and bad things. It didn’t make me feel bad and it’s good to show that.

### Text/SMS

#### Text Messages Were Helpful

Overall, patient-participants found the text messages easy to understand, helpful, and encouraging. One patient-participant said that the messages provided information she would not be able to get from her friends. When asked what they thought about the text messages, one patient-participant said:

A lot of them were helpful actually. The eating, changing the diet, getting a car seat immediately, the exercising and taking showers when you feel like you want to smoke.

The most common suggestion was to include more resources, either specific resources from the community they felt were missing or more tips on how to quit. When asked about frequency, 3 patient-participants said they would like to receive texts once a week, 3 said twice a week, and 4 said 3 or more times a week.

#### Learned About Community Resources

Many patient-participants learned about community resources they were not aware of and found the additional tips on ways to avoid temptation or keep from using helpful. When asked what she thought about the messages, one patient-participant stated:

They were helpful. There were not only tips but there were resources and phone numbers.

Another response suggested adding even more information about resources.

Add more about the resources. 2-1-1 and WIC are good, maybe [add] FIA (Family Independence Agency/Department of Human Services) info.

#### Information Presented Was Overwhelming

In total, 4 participants indicated that there was too much information presented in the text messages. However, this concern appeared to be related to the way it was presented (1 message for each week of pregnancy, all in one document) and not the amount within each individual text message.

### Provider-Participant Focus Groups

#### Concern About Patient Takeaway

Both doctors and staff reported liking the intervention overall. Many commented on the strength of the message. Some felt there needed to be more emphasis on the dangers of marijuana use and the need to completely quit. Overall, both focus groups understood and appreciated the positive message behind “the less you use the better” for both the woman and the baby. Provider-participants felt the doctor was very relatable and felt like a real doctor rather than an actress.

#### Integration Into Patient Visit

Doctors and staff agreed that overall the message was important for their patients to hear and that the information provided and actresses chosen would be relevant for their patients. Provider-participants reported that there may be several junctures at which to incorporate the program into the flow of the patient visit. For example, they indicated that the point after the patient is brought to the exam room, but before the patient is seen by the physician, is generally wasted. They also felt that the 26-28 week glucose screen all pregnant patients must complete would be another opportunity to present the intervention (or a follow-up intervention) because the patient is required to wait for 1 hour before the blood is drawn.

#### Quality of the Presentation

Although provider-participants generally liked the actresses, there was one actress in particular that staff felt was not natural or relatable (the same actress criticized by the patient-participants). Half of the medical staff did not like the voice for the animated character. Other specific suggestions included decreasing the amount of information presented on the screen at one time and cutting back on the length of the introductions and transitions during the intervention. Medical staff suggested changing the order of the text messages to fit better with the stage of pregnancy.

## Discussion

### Principal Findings

This study sought to evaluate, among pregnant women and prenatal care providers, the acceptability of an electronic brief intervention and text messaging program to reduce marijuana use in pregnancy. Following the technology acceptance model [30], we evaluated participants’ acceptance of the interventions, video and text messaging, and their perceived usability and ease of use. Overall, patient-participants rated the brief intervention positively, more specifically as helpful, respectful, interesting, and easy to use. The videos that were embedded within the brief intervention were also seen as unbiased and as presenting helpful information about cessation and the effects of marijuana on the baby. The information was well received, although one actress for the testimonials was consistently given negative feedback. Similarly, the text messaging content was seen as helpful in providing information about community resources and additional tips on how to reduce or cease using marijuana during pregnancy.

Provider-participant feedback was similarly positive regarding the brief intervention content. They saw the information as both relevant and important for their patients to hear. However, questions were raised regarding the strength of the message and whether it should focus on complete cessation or harm reduction. They offered several suggestions regarding integration into existing structure of patient care, including utilizing the time between intake procedure and examination and follow-up or initiation of intervention during the mandatory glucose screening visits.

These findings suggest that the women in this study were open to examining their marijuana use during pregnancy and to doing so via technology. Participants were happy with the unbiased presentation of the effects of marijuana on the baby, found the materials useful and easy to use, and clearly spent time evaluating whether or not they should stop use during pregnancy.

### How Feedback Informed the Planned Clinical Trial

On the basis of the feedback from both patient-participants and provider-participants, several changes were made to the intervention materials in preparation for the planned clinical trial. The order of the information presented in the text messages was improved based on the stage of pregnancy (ie, suggestions for dealing with nausea were moved to the beginning when women are more likely to be struggling with morning sickness). Additionally, the amount of text on screen and introduction language used during the intervention was reduced to only the key pieces of information. Not all feedback provided by provider-participants and patient-participants could be incorporated into changes to the intervention. Changes to the voice of the narrator were not possible. However, to facilitate more consistently positive feelings toward the narrator, research staff carefully reviewed the narrator actions changing narrator movements and using less smiling.

On the basis of the feedback from provider-participants, future clinical trial participants will be recruited, screened, and will participate (using a tablet provided by study staff) during natural periods of downtime during their prenatal visit (eg, while the patient is waiting to see the provider, in the exam room, or immediately after the visit is complete). Furthermore, based on the feedback from patient-participants regarding the frequency of receiving text messages, those enrolled in the clinical trial will be offered the choice of receiving text messages on their personal phone once, twice, or thrice per week for the remainder of their pregnancy. Despite some patient-participants reporting little use of texting, all reported the ability to receive text messages. Finally, recruitment rates obtained during feedback suggested that alternate recruitment options (such as partnering with midwifes and ultrasound technicians) should be considered.

### Limitations

This study is limited by its relatively small sample size of all African American women from a clinic in the urban Detroit area. However, our aim was not to conduct a fully powered test of an a priori hypothesis, but rather to provide information regarding participant acceptability and usability, which typically involves smaller sample sizes. Additionally, it may have been preferable to present text messages for feedback as a presentation where each text message could be looked at separately. Having a single document with a sample of each week’s messages was overwhelming for some participants. Future studies should also consider ways to tailor the text messages for the participants providing feedback. This study was only able to show examples and describe how messages would be tailored and may have missed valuable feedback because of the presentation format.

### Conclusions

Technology-based approaches have the potential to access a relatively high proportion of any given at-risk population. If this potential is realized, it could result in a substantial public health impact even when effects are modest. Furthermore, promotion of help-seeking is also an integral part of the proposed high-reach interventions, making them an ideal complement to more intensive support programs, where available. The acceptability (and even preference in some cases) of these technology-based approaches for marijuana use in pregnancy suggests that further research, particularly evaluation of efficacy, is needed.

## Abbreviations

eSBIRT: electronic administration of SBIRT
IRB: institutional review board
PC: personal computer
SBIRT: screening, brief intervention, and referral for treatment
